# Highlights from the 15th St Gallen International Breast Cancer Conference 15–18 March, 2017, Vienna: tailored treatments for patients with early breast cancer

**DOI:** 10.3332/ecancer.2017.732

**Published:** 2017-04-07

**Authors:** Consuelo Morigi

**Affiliations:** Division of Breast Cancer Surgery, European Institute of Oncology, Via Ripamonti 435, 20146 Milano, Italy

**Keywords:** 15th St Gallen Consensus Conference 2017, early breast cancer, tailored treatments

## Abstract

The 15th St Gallen International Breast Cancer Conference was held in Vienna for the second time, from 15th–18th March 2017. 4000 people from 105 countries all over the world were invited to take part in the event. The real highlight of the conference was the last day with the International Consensus Session which was chaired by around 50 experts on breast cancer worldwide. With reference to data from scientific research, the consensus panel tried to offer guidelines for the management of breast cancer with the aim of providing patients with optimal treatment. The topics covered focused on the treatment of breast cancer, consideration of surgery, radiotherapy, neo-adjuvant, and adjuvant systemic therapy for breast cancer, as well as genetics and prevention of breast cancer.

In particular, in terms of precision medicine, an important topic of the conference was ‘is it possible to think that it could become routine in clinical practice to use immunotherapy and targeted therapy based on genetic signatures?’

In view of personalised therapy, it is important to take into consideration women’s treatment preferences. It is also important not only to offer guidelines which help breast cancer experts all over the world to choose the proper treatment for women with breast cancer but also to discuss the pros and cons of the therapy with the patient. This allows for a better understanding of the disease.

‘From the maximum tolerable to the minimum effective treatment: it is essential to escalate treatment when necessary and to de-escalate when unnecessary’. These few words could summarise the meaning of the 15th St Gallen International Breast Cancer Conference. Prof Martine Piccart-Gebhart was awarded with the St Gallen International Breast Cancer Award 2017 for her fundamental clinical research contribution and Prof Giuseppe Curigliano with the Umberto Veronesi Memorial Award which aims to recognise a physician’s leading role in advancing the science and care of breast cancer patients. Curigliano, in his lecture, spoke about the revolutionary immunotherapy in the clinical management of breast cancer (BC). For the development of these therapies, it is necessary to identify the genetic determinants of BC immune phenotypes in which The Cancer Genome Atlas (TCGA) has contributed towards this.

For example, the T helper (Th-1) phenotype (ICR4), which also exhibits upregulation of immune-regulatory transcripts (eg. PDL1, PD1, FOXP3, IDO1, and CTLA4), was associated with prolonged patients’ survival. Chromosome segment 4q21, which includes genes encoding the Th-1 chemokines CXCL9-11, was significantly amplified only in the immune favourable phenotype (ICR4). The mutation and neo-antigen load progressively decreased from ICR4 to ICR1 but could not explain immune phenotypic differences. Mutations of TP53 were enriched in the immune favourable phenotype (ICR4). Instead, the presence of MAP3K1 and MAP2K4 mutations were closely associated with an immune unfavourable phenotype (ICR1). Using both the TCGA and the validation dataset, the degree of MAPK deregulation segregates BC according to their immune disposition. These findings suggest that mutational-driven deregulation of MAPK pathways is linked to the negative regulation of intratumoural immune response in BC.

The main themes of this congress were: 1) Surgery of the primary tumour and margins; 2) Surgery of the axilla; 3) Radiotherapy: hypofractionated, ‘boost’ to tumour bed, partial breast, regional node, after mastectomy, advanced technology; 4) Pathology: subtypes, TILs; 5) Multi-gene signatures and therapy; 6) Endocrine therapy: pre- and post-menopausal and duration; 7) Chemotherapy: subtypes, stages; 8) Anti-HER-2 therapy; 9) Neo-adjuvant therapy; 10) Adjuvant bisphosponates; 11) Adjuvant diet and exercise.

## News Since Last St Gallen: De-Escalating and Escalating Treatment

M Piccart-Gebhart introduced this important topic. She demonstrated its relevance and importance in all areas of BC treatment.

M Marrow opened the scientific session with a summary on the margins consensus in BC which according to SSO-ASTRO-ASCO should be at least 2 mm with a clinical judgement in determining whether patients with smaller negative margins require re-excision in DCIS taking into account: residual calcifications, life expectancy, extent of DCIS near margin, cosmetic impact of re-excision, adjuvant therapy. Regarding axillary lymph node dissection (ALND), it appears necessary to select the approach which minimises the need for it, and so the neoadjuvant therapies in N+ and clinical practice of trials like Z0011 intraoperatively have become important.

Regarding de-escalating and escalating in radiotherapy (RT), as stated by P Poortmans, it is necessary to consider the life expectancy of patients, comorbidities, define the tumour risk (stage, biology) and share decision-making benefits/risks with patients. We can consider for example avoiding RT in low risk and older women with a short life expectancy [1].

I Smith stated that even in chemotherapy, we must consider it for every single patient and then choose the correct approach without redundant or underdose therapy: Sometimes an escalating therapy is permissible, as for example in some patients at high risk for whom we can extend adjuvant endocrine therapy after five years, especially after five years of tamoxifen (NSABP B-42, DATA). Also, an addition of capecitabine in triple negative breast cancer (TNBC) causes survival benefits (FinXX, CREATE-X). On the other hand, sometimes it is advantageous to de-escalate treatments, for example in patients with moderate risk we probably do not need anthracyclines (ABC trial); four courses of chemotherapy (with AC or paclitaxel) are as good as more in OFS and OS according to data [2], 5 FU is contraindicated in early breast cancer not increasing overall survival (OS) or disease-free survival (DFS) but resulting in increased side effects (neutropaenia, fever, nausea, vomiting) [3], the neoadjuvant approach, in some patients, may offer a possibility of reducing the intensity of treatment (for example in HER-2+, according to the APT, NeoSphere, and KRISTINE trials).

M Regan discussed the role of trial designs in supporting treatment escalation and de-escalation. For example, the SOFT trial justifies suppressing ovarian function in pre-menopausal women with high risk; two innovative trials, including multi-gene assays, TailorX and Mindact, provided clinical evidence to identify patients for de-escalation of chemotherapy.

## Genetics and Prevention in Breast Cancer:

This part of the conference was chaired by J Garber, and it was focused on the importance of genetic signature in BC. By introducing next generation sequencing, clinical laboratories can efficiently examine many genes concurrently enabling an assessment of other BC susceptibility genes in related DNA repair pathways or clinical syndromes in addition to well-known BRCA1/2 mutation carriers. With the introduction of next generation sequencing, it is possible to examine many genes concurrently, enabling the assessment of other BC susceptibility genes in related DNA repair pathways or clinical syndromes with several benefits, such as: more complete identification of underlying inherited predisposition in individuals and families previously undefined, enhanced assembly of mutation carriers in genes less well studied, accelerated clinical trials examining agents targeting DNA-repair defects (eg. ALB2, CDH1, CHEK2, NF1, TP53, ATM) [4]. Clinical interpretation of this data set is not simple (the presence of somatic mosaicism or of other low/moderate penetrance genes): Many studies are needed to test the safety of these approaches because their results could have a significant impact on women’s lives in terms of anxiety and psychological problems.

The remaining part of the session focused on several preventive treatments in women with breast tumours and was led by P Karlsson, J Cuzick, and A Partridge. There are a few factors which support the use of postoperative RT after ductal carcinoma *in situ* DCIS, including: premenopausal status, high nuclear grade, presence of necrosis, preference of patient, absence of important comorbidity such as cardiovascular diseases or diabetes, availability of gated RT. Many trials have studied the effects of postoperative radiotherapy after breast-conserving surgery in patients with DCIS, and they have shown that radiotherapy halves the risk of ipsilateral events without, however, having any significant effect on breast cancer mortality (SweDCIS, RTOG9804). It has been challenging to gain knowledge of the natural course of DCIS and identification of subgroups without the relative efficacy of radiotherapy. Recent trials have demonstrated that five years of tamoxifen produce a very long-term 30% reduction of BC and that the benefits of this treatment continued for at least 20 years after starting it (IBIS trial). The most important side effects of this drug are thromboembolism and endometrial cancer. In comparison with tamoxifen, raloxifene was found to have fewer of these side effects, but with less efficacy. Aromatase Inhibitors (AI) are more effective with a reduction of 50% of BC (IBIS-II, MAP3) and less toxic, but they are suitable only for postmenopausal women; this superiority of AI seems to appear also in DCIS (NSABP-B35 and IBIS-II (DCIS). Further trials are needed to explore new preventive treatments for premenopausal women. In the context of the prevention of invasive breast cancer, researchers are actively working to determine if DCIS can be managed safely without surgery (e.g. the COMET and LORIS trials), but to make this possible it is very important to improve communication with patients and to remove the term ‘carcinoma’ from DCIS. In fact, the use of non-medical and clearer language can determine a shift in women’s treatment preferences from surgical to non-surgical treatments, suggesting that many women may prefer a less invasive, non-surgical option if allowed to weigh each choice and its attendant risks.

## Targetable Pathways in The Clinic

A Tutt stated that targeting DNA repair deficiencies in cancer has many important clinical benefits. DNA repair defects and recombination signal are promising targets; in fact they leave distinctive mutational scars and may cause transcriptional signatures that need validation as biomarkers and therapeutic targets.

An example is represented by breast cancers BRCA1/2-mutated and some sporadic TNBC that have DNA repair defects, and they are sensitive to DNA-damaging drugs like platinums and Poly (ADP-ribose) polymerase (PARP) inhibitors. A weighted model called HRDetect was developed to accurately detect BRCA1/BRCA2-deficient samples. HRDetect identifies BRCA1/BRCA2-deficient tumours with 98.7% sensitivity (area under the curve: 0.98). Application of this model allowed the identification of additional tumours with alteration of BRCA1 or BRCA2, which could have selective therapeutic sensitivity to PARP inhibition [5, 6].

J Baselga reported on the clinical utility of P13K inhibitors. P13CA is the most frequently mutated gene in HR+ BC. Unlike older P13K inhibitors, the next generation of this drug has an increased selectivity against P13Kalfa and consequently fewer side effects. An example is represented by Taselisib which has enhanced potency in P13CA mutant models because it uniquely degrades mutant p110a protein, and it continues to be investigated in ongoing clinical trials (Sandpiper); SOLAR-1 is another phase III study that is underway and investigates the role of another P13Kalfa inhibitor, alpelisib.

P Goodwin stated that regarding lifestyle interventions like weight loss and enhanced physical activity, we know that obesity is associated with a modest increased risk of poor BC outcomes, and that physical activity is associated on the other hand with modest BC outcomes. Though currently there are no data supporting the benefit of lifestyle habits in improving BC outcomes [7]. However, there are ongoing trials (e.g. SUCCESS, DIANA 5, BWEL) that will provide definitive evidence regarding an impact on BC.

## Biology

In this session the authors focused on the important implications of clinical assays for making treatment decisions of intratumoural heterogeneity (ITH).

LR Yates, based on current evidence, stated that most driver alterations in the primary tumour denote a subclone origin of aggressive clinical behaviour such as those seeding metastatic dissemination or treatment resistance [8]. The results, in order to prevent relapse, provide a rationale for basing adjuvant therapies on the primary tumour sample. In this context, the target should be to incorporate well-designed clinical-genomic studies into all clinical trials.

C Perou revealed the existence of intra-tumour subclonality on multiple levels including DNA, RNA, protein, and epigenetic cellular states. In reference to this consideration, the measurement of subclonality before therapy can provide additional predictive or prognostic information about treatment response and survival. Equally important, given the fact that they have a different behaviour, is the measurement of subclonality after therapy. In fact, unlike DNA copy number in which changes tend to be determined early, these are maintained throughout progression and only showing slight differences within a tumour. Somatic mutations happen early but also late in tumour progression and can show significant ITH [9]. Remarkable recent examples of this include ESR1 mutations that result within the setting of aromatic inhibitor treatment.

F Andre in his presentation reported that there are many repeated targetable genomic alterations that could be correlated with drug sensitivity. Among all, we can quote mutation of PIK3CA associable with sensitivity to alpha-selective PI3K inhibitors, mutations of AKT1, and ERBB2 associable with sensitivity to AZD5363, and neratinib, and mutations of BRCA2 linked with sensitivity to poly ADP ribose polymerase (PARP) inhibitors. Moreover, there are also rare genomic alterations. Consequently, the rationale to test a large panel of genes in patients with cancer treatment resistance is justified. Even though phase III studies at the moment do not show evidence that using a large panel of genes improves outcomes, ongoing trials are still testing this hypothesis. In patients with metastatic cancer, a valid alternative to biopsy in order to discover genomic alterations could be circulating DNA associated with drug sensitivity. This treatment strategy offers us many advantages: first of all the procedure is not invasive, secondly it represents the whole genomic heterogeneity in each patient, and thirdly it could identify therapy resistance early prior to imaging [10].

D Hayes addressed the issue of clinical utility in the adjuvant setting of several tumour biomarker tests. Multi-parameter assays (Oncotype, Endopredict, Prosigna, BCI, and perhaps Mammaprint) have demonstrated validity and clear clinical utility for prognosis in low risk breast cancer (ER positive, HER-2 negative, node negative). It is shown that patients with a low composite score have a favourable prognosis and the balance between benefits and risks does not justify the use of chemotherapy. Instead, the topic of adjuvant therapy in terms of duration of the endocrine therapy (ET) remains complicated. If on one hand it is true that the majority of deaths in ER positive breast cancer occur after five years and extended ET reduces this risk by 1/3 –1/2, on the other hand extended ET has several side effects. The goal is to have better prognostic factors to decide who are the ideal candidates to receive extended ET, despite the fact that no genomic assays had adequate analytical validity or clinical utility to recommend their use in guiding extended ET [11].

The main point of the presentation of K Osborne was a possible near future without chemotherapy for HER-2+ breast cancer. During his speech he spoke about ‘dual blockade’, a more complete blockade of the HER receptor layer. Therapies targeting HER-2 such as trastuzumab are known to be very effective since a long time. Even if these therapies have been given traditionally in association with aggressive chemotherapy, there are instances when many neoadjuvant clinical trials (e.g. TBCRC 006, TBCRC 023) with combinations of HER inhibitors like lapatinib or pertuzumab with trastuzumab have shown effectiveness without chemotherapy. Some tumours present resistance to this approach because of for example, mutations in PIK3CA or low levels of HER-2.

Actually, there are ongoing validation studies of this and other markers. If they are validated, they may be used in selected patients for targeted therapies without chemotherapy in HER-2+ breast cancer.

The acquired resistance to ET is a problem that oncologists are facing every day. It is known that there are multiple oncogenic drivers and resistance pathways in HR+ BC, and that combined blockade of both driver pathways and resistance mechanisms can be synergistic. Regarding this topic, I Krop, discussed mutations in the gene encoding ER (ESR1) which are associated with resistance to AI [12]. Preliminary data suggested that agents degrading ER like fulvestrant or selective oestrogen degraders (SERDS) may overcome this mechanism of resistance (GDC-810). In a similar way, the addition of the mTOR inhibitor everolimus to exemestane or fulvestrant can block the crosstalk between ER and the PI3K/mTOR pathway that has been implicated in hormonal therapy resistance. If on one hand inhibitors of all isoforms of P13K-like buparlisib have been disappointing in terms of benefits, on the other hand PI3K inhibitors selective for the mutated isoform such as alpelisib and talselisib according to current phase 3 trials have shown a greater therapeutic index particularly against PIK3CA mutant cancers. BC with mutations in the gene encoding HER-2/ERBB2 may be susceptible to potentiate HER-2-kinase inhibitors such as neratinib.

S Loi presented the complex argument of resistance to chemotherapy of Triple Negative Breast Cancer (TNBC). Genomic analyses have revealed that TNBC has genomic instability with a loss of function in gatekeeper genes and the amplification of oncogenes resulting in tumour growth, adaption to treatment, and drug resistance. It is quite difficult to imagine that single targeted therapies will be the solution for TNBC. To win this challenge we need a combination of more factors like more powerful DNA damaging agents, immunotherapy challenging genome instability, and antibodies conjugation.

## Pathology

S Schnitt reported that there is no general agreement nowadays regarding the form of an adequate negative margin in conserving breast surgery. The SSO-ASTRO-ASCO consensus margin guidelines of 2 mm in conserving breast cancer are designed to help to standardise the practice without replacing clinical judgment. In particular, they are not to be applicable to patients treated with partial breast irradiation, lumpectomy without whole brain radiation therapy (WBRT), neoadjuvant systemic therapy, and in patients with DCIS. Regarding these DCIS patients treated with breast conserving surgery and WBR, the routine practice of obtaining margins wider than 2 mm is not supported by any evidence. For all, adoption of these guidelines has the potential to reduce re-excision rates with, more importantly, a decrease in patient’s anxiety, morbidity, cost, adverse effect on cosmesis, and a delayed start to systemic therapy. The consensus panels recognised that anyhow there are circumstances in which margins wider than no ink on the tumour may be indicated for patients with invasive cancer and in which margins less than 2mm may be acceptable for patients with DCIS [13, 14, 15].

C Sotiriou remarked that an adequate estimation of patients’ prognosis is crucial to avoid overtreatment and to help optimisation of their adjuvant management strategies. Patients classified as ‘equivocal cases’ by classical clinical pathologic features (like women with ER+, HER2neg, N0, grade 2, Ki67 between 10–20%), and the integrating multi signatures (eg. EndoPredict, Genomic Grade Index, PAM50, Breast Cancer Index, Oncotype, DX, MammaPrint) for this reason can be used to help decision-making on treatment.

G Viale focused on the controversial treatment of patients with equivocal expression of ER and HER-2. ER-poor BC are tumours in which 1–9% of tumour cells express ER. These tumours are rare, so prospective clinical trials have not been conducted. Consequently, the optimal treatment strategy is based upon the data of individual institutes or retrospective analysis of randomised clinical trials. Long-term prognosis of ER-poor tumours is controversial with some studies reporting clinical outcomes similar to those of ER-negative cancers, and others showing an intermediate prognosis compared to ER-negative and ER-rich tumours. Whether these tumours are responsive to endocrine drugs or not is still a big question mark, but since a potentially life-saving benefit from empirical adjuvant ET cannot be excluded, the clinical approach of these BC may be to consider both adjuvant ET and chemotherapy. According to the current ASCO/CAP guideline recommendations, an *in situ* hybridisation test for HER-2 is equivocal when the mean gene copy number is between 4 and 6, and the gene on chromosome 17 ratio is less than 2. If the initial HER-2 test result is equivocal, reflex testing should be performed on the same specimen using an alternative test. If the HER2 test result is considered equivocal again, one may evaluate for HER2-targeted therapy. Nowadays, the benefit of HER-2-targeted therapies for these patients are unknown. A randomised phase III trial, NSABP B47 will probably clarify the optimal treatment strategy for patients with a low or equivocal HER-2-expression.

C Denkert presented a subgroup of BC characterised by a dense tumour infiltrating lymphocytes (TILs), which is particularly significant in TNBC and HER-2+ BC. TILs is demonstrated to have a good prognosis in TNBC and HER-2+ BC. It has been described as a predictor of pathological complete response to chemotherapy in many prospective neo-adjuvant clinical trials and its increase appears linked to an improved prognosis after adjuvant therapy. TILs can be used as a prognostic marker as shown in a variety of clinical trials (eg BIG-2-98, FinHER, Cleopatra), providing a typically 15–20% improved survival per 10% increase in TILs.

N Disis also focused her lecture on the immunogenicity of BC and the important role of TILs. In conclusion, it might be useful for clinical decisions in selected patients and should be included in pathology reports.

## CDK 4/6 Inhibitors: When? Why? Who?

The therapeutic role of CDK4/6 inhibitors was particularly investigated throughout the congress, during both the scientific sessions and the numerous sponsored symposia such as from the first one in chronological order i.e. the symposium organised by *e*cancer. Cyclin D1-CDK 4/6-retinoblastoma pathway is a regulator of the cell cycle, in particular it controls the checkpoint for entering into the S-Phase. Inhibitors of CDK 4/6 are important in contrasting the amplification of CDK4/6 and overexpression of cyclin D which are very common in BC.

The many physicians who spoke about this important theme (G Curigliano, S Loibl, G Shubassi, P Earl, C Criscitiello, M Gnant, S Delaloge, L Gianni) were unanimous in supporting the use of these drugs (RIBOCICLIB, ABEMACICLIB, PALBOCLIB,) in women with mBC HR+, HER-2-, resistant to ET. These medicines, which are similar in efficacy but differ in dosage and toxicity, have shown overall acceptable safety and tolerability. Among the main side effects we can emphasise on reversible neutropaenia with the discontinuation of the drug, nausea, diarrhoea and vomiting are pharmacologically manageable. Numerous trials (eg PALOMA 1/2/3, MONALEESA-2) have shown an increase in progressive free survival (PFS) by adding these drugs to endocrine therapy.

Abemaciclib as monotherapy in heavily pretreated mBC patients demonstrated an impressive 19% overall response rate. It is under investigation in phase 2 and 3 in combination with a non-steroidal aromatase inhibitor (MONARCH 3, NCT024 6621) and fulvestrant (MONARCH 2 NCT021007703). The NeoPalAma trial has shown a significantly more complete cell cycle arrest defined as Ki67≤2.7%) after adding palbociclib to anastrozole. There are many ongoing trials that test the association of CDK4/6 inhibitors to trastuzumab, associated or not with ET in breast cancer HER-2+ (monarcHER, PATRICIA, CLEE011XUS20T, NCI-2016-00626, STU 042013-042).

## Neo-Adjuvant and Adjuvant Systemic Therapy

N Harbeck focused her presentation on the neo-adjuvant chemotherapy in TNBC and in HER-2+ tumours. Achievement of pathological complete response (pCR) at the time of surgery is correlated with a positive patient outcome. Anthracyclines associated with Taxanes is actually the standard neo-adjuvant therapy in TNBC, but in the meantime there are many studies investigating more effective therapies. The use of nab-paclitaxel for example instead of paclitaxel has demonstrated a significant improvement of the pCR rate (GeparSepto). The addition of platinum to anthracycline and taxane improved the pCR rates (CALGB 40603). In HER-2+ EBC, a dual blockade with trastuzumab and pertuzumab associated with chemotherapy has been approved by European Medicine Agency (EMA) and Food and Drug Administration (FDA) for neo-adjuvant therapy (NeoSphere, CLEOPATRA). Finally, in the TNBC sub-trial data with 12 weeks of neo-adjuvant nab-paclitaxel and carboplatin, when seen in terms of pCR (breast and axilla) was >40%, and in the HER-2+ sub-trial, the pCR was about 90% with 12 weeks of paclitaxel weekly plus dual blockade (WSG ADAPT, Umbrella). Unfortunately, neo-adjuvant trials have usually been too small to statistically prove a survival advantage. Future research needs to focus on avoiding overtreatment of patients with pCR as well as on improving therapy options for patients with non-pCR after neo-adjuvant treatment. Here, early response markers (e.g. biomarkers, molecular imaging) and also novel targeted agents may play an important role in the future.

Neo-adjuvant ET in postmenopausal women with ER+ stage II/III tumour is currently underused, although it shows low toxicity. Improvements in surgical outcomes are not the only advantages of this approach, since each patient can be closely monitored for treatment response in order to make a judgment regarding optimal management. Many trials i.e. Z1031A, ALTERNATE and P024 seem to show the advantages of using AI in neo-adjuvant setting as presented by M Ellis.

P Francis focused attention on adjuvant ET. The use of adjuvant TAM for five years in premenopausal women according to data from meta-analysis has been demonstrated to have substantial reductions in recurrence with an important improvement of overall survival (OS). This treatment is particularly indicated in women >45 yrs because of its safety profile. In women allocated to five years of TAM at age <45 yrs, the risk of contralateral BC highlighted an absolute reduction of 2.9%. According to the ATLAS trial, continuing TAM up to ten years rather than stopping at five years produces an addition to a statistically significant improvement of OS; also a further reduction in recurrence after year seven and in BC-specific mortality (after year ten) with an absolute benefit of about 4% for recurrence and of about 3% in BC-specific mortality. The initial results from the Suppression of Ovarian Function Trial (SOFT) and TAM and exemestane Trial (TEXT) evidenced a statistically significant reduction in BC recurrence with the association of ovarian function suppression (OFS). It has been seen that especially the use of exemestane + OFS may provide a large absolute increase (10–15%) in DFS, and it has proven to be a more effective therapy than TAM + OFS, or TAM alone.

HJ Burstein and M Dowsett reported on adjuvant ET in postmenopausal women in their presentation. Treatment options include TAM, AI, or a sequence of these two drugs. Individual trials and meta-analysis suggest that AI treatment, as either initial or sequential therapy, reduces recurrence risk compared to five years of TAM alone. Until today, there has been no unique reference biomarker for every treatment; however, women with tumours that have higher risk features may obtain more significant benefits from AI treatment and even outcomes for lobular carcinomas may be better.

Because of the fact that adjuvant ET reduces breast cancer recurrence, women with very small (less than 1 cm) node negative invasive cancers have also taken advantage of this treatment.

Data derived from recent trials showed a steady risk of BC recurrence for at least 15 years after five years of ET. Extending ET (NSABP B-42, IDEAL, DATA) surely reduces the risk of recurrence. In any case considering that these drugs also have side effects, the aim is to identify which patients have sufficient residual risk to potentially benefit from this extension. According to prognostic signatures, clinical treatment score, immunohistochemical markers, Oncotype recurrence score, Prosigna, Breast Cancer Index, and Endopredict, the risk of relapse is different and consequently more data are needed to predict better residual risk of recurrence. Currently we should consider extension of ET in women with high stage BC, especially patients who have tolerated the treatment and are willing to continue, and patients who started with TAM.

H Joensuu spoke about systemic treatment in HER-2-positive breast cancer. Nowadays adjuvant standard therapy in these tumours consists of chemotherapy plus 12 months of trastuzumab. Modifications of this standard have been investigated by several randomised trials like for example NeoALTO, NSABP B-41, TRIO-US, CHER-LOB, KRISTINE. These have compared dual HER-2 inhibition plus chemotherapy (consisted of trastuzumab plus either lapatinib, neratinib, or pertuzumab) with trastuzumab plus chemotherapy as neoadjuvant treatments for early HER-2-positive breast cancer. In all trials, the combination of dual HER-2 inhibition plus chemotherapy provides a numerically higher pathological complete response (pCR) rate. Other trials focused attention on the modification in duration of trastuzumab administration in the adjuvant setting. The results showed that patients subjected to the treatment of adjuvant trastuzumab, with less than 12 months, did not have statistically importantly inferior DFS or OR (eg. PHARE) [16]. However, these three trials have critical issues in that the results favoured numerically the 12-month treatment in two of them; two of these trials were small, and one had a comparatively short follow-up time of the patients. HERA and ExteNET, two randomised trials, have evaluated longer than one year of adjuvant HER-2-targeted therapy. In the HERA trial, the extension of >one year of adjuvant anti- HER-2 therapy has not showed differences in DFS and OS but surely two years of treatment were more cardiotoxic [17]. In the ExteNET trial, extending >one year adjuvant trastuzumab with one year of neratinib, the outcomes revealed an improvement in DFS in the subset of patients with HER-2-positive and ER-positive cancer even though no OS benefit was reported, and the patient’s follow-up time was still too short. The results of many ongoing trials ( eg. ALPHINITY, KATHERINE, KAITLYN, BOLD-1) may modify the current standard treatment.

L Carey stated the possibility of an increased tailoring treatment in TNBC. It looks very interesting that even in this high risk tumour we can consider a de-escalating in treatment with a reasonable omission of chemotherapy like for example in T1a/T1b, N0 tumours. Regarding the escalation, large neoadjuvant studies (GeparSixto, CALGB 40603, ADAPI-TN) demonstrated a notably increased pathologic complete response (pCr) with the addition, beyond anthracyclines and taxanes, of platinum in TBC. Nevertheless, the impact on clinically-significant outcomes remains unknown. At the present time it is better, in a residual disease high-risk setting after neoadjuvant chemotherapy, to use only capacitabine (CREATE-X trial). Even if not yet validated, biologic understanding helps us to escalate and de-escalate therapy in TNBC. In FinHER, for every 10% increase in stromal tumour infiltrating lymphocytes in the TNBC subset, there was a nearly 25% improvement in DFS; and in CALGB 40603 pCR was highly associated with activated immune gene signatures.

Di Leo started focusing the necessity of accurate prognosis allowing the estimation of those patients who are already cured by loco-regional therapy and those who require adjuvant therapies. There are many pathologic and genomic instruments where in on one side they may overestimate the risk of disease relapse and on the other side instruments that identify the presence of micro-metastatic disease might help us in refining prognostic assessment. A potential tool to predict the outcome in patients with early breast cancer can be circulating tumour DNA (ct DNA). The use of H-NMR based spectroscopy on the biological fluid allows us to measure hundreds of metabolites at the same time (metabolomic spectra). The necessity of clinical flexibility, nowadays, is fundamental according to the fact of how uncertain it is tailoring the different chemotherapy options to each individual case. As a conclusion, Di Leo declared that in Luminal A-like tumours, newer or older treatments have approximately the same evidence. In Luminal B-like tumours, anthracyclines + taxanes is a well-designed treatment despite the fact that no evidence is given based on data concerning dose-density regimens by luminal sub-types. It seems to be possible that through meta-analysis in the near future we will focus the ideal guidelines for treating our patients.

M Gnant, as shown in a recent meta-analysis (EBCTCG), stated that bisphosphonates in the adjuvant setting play an important role.

First of all, bisphosphonates prevent bone reabsorption, secondly they prevent reduction in bone mineral density and the risk of fractures resulting from oestrogen (or androgen) deprivation in cancer therapies, and lastly they reduce bone recurrence and improve survival in postmenopausal women with non-metastatic breast cancer (no significant effects on non-breast cancer deaths, contralateral breast cancer, or loco-regional recurrence).

According to the ‘low-oestrogen’ hypothesis, bone-modifying adjuvant treatments silence the bone marrow microenvironment in a certain way. Because of that reason, relevant clinical benefits are demonstrated in terms of reducing relapse and breast cancer deaths. These are seen only in postmenopausal patients or in premenopausal patients on ovarian function suppression therapy. Denosumab also reduces the risk of clinical fractures in postmenopausal breast cancer women receiving AI, but it is not yet known if it reduces the risk of breast cancer recurrence too. The only way to prove this hypothesis is looking through large individual patient level meta-analysis (ABCSG-18, D-CARE) through collaboration with treating physicians. Their role is to explain these ongoing studies and the potential benefits and risks from the use of these drugs. Based on these results, these drugs may be licensed for use in the adjuvant setting and in the absence of osteoporosis [18, 19]

## Surgery

V Galimberti placed the focus on the oncological safety of nipple-sparing mastectomy (NSM) [20]. She spoke on how allowing the preservation of most of the breast skin and nipple-areola complex (NAC) consequently improved aesthetic outcome. This in turn had improvement in patient satisfaction and psychosexual benefit. The NSM is a safe surgical procedure even in patients undergoing neo-adjuvant chemotherapy, and they are also suitable in prophylactic mastectomies in high-risk women [21, 22]. For its feasibility, it is a mandatory negative retro-areolar frozen section. Nevertheless, the main complication of NSM is necrosis of the NAC. Data indicate, however, that as surgeon experience increases, the frequency of this complication declines. A new technique is represented by robotic NSM that, in selected patients, is reliable and reproducible with a brief learning curve [23].

JT Rutjers focused his presentation on the intraoperative assessment of sentinel lymph node (SLN). The SLN procedure was changed by the Z0011 trial, i.e., they included women undergoing conservative breast surgery and WBRT who have 1–2 metastatic SLN should not have received axillary lymph node dissection (ALND). More controversial is seen in the approach of patients undergoing mastectomy. In these women many surgeons continue to perform assessment of SLN, nevertheless these patients in case of positive SLN, according to EBCTCG overview, seem to benefit from RT. Intra-operative assessment of the SN can be used in patients with proven lymph node metastasis at primary diagnosis and who have been treated with neo-adjuvant chemotherapy. In these patients, ALND is always considered the standard of care, but if the SLN (and or clipped node) after upfront therapy is free of disease, ALND could be avoided. It is imperative that decisions about how to treat axilla is made after multidisciplinary consultation (surgeon, oncologist, radiotherapist) with knowledge of tumour biology, burden disease in the sentinel nodes, planning of possible radiotherapy, and systemic therapy, as well as without omitting patient preferences. The main advantage of intraoperative assessment of axillary lymph nodes is that metastatic disease can be diagnosed and removed in a single operation, but to prevent the increase of false positive or false negative results, intraoperative evaluation should be sometimes more accurate. [24, 25, 26]

W Weber, an expert in oncoplastic surgery (OPS), stated that currently there are no randomised controlled trials to support the efficacy and safety of this surgical procedure, but prospective and retrospective observational studies show an oncological safety of the widespread use of OPS.

What impacts surgical decisions? T King declared that for both age and subtype, the intrinsic biology of the tumour (most often aggressive in younger patients) is the strongest predictor of the outcome [27]. Recent data, linked to improving local therapy strategies ( e.g. negative surgical margins, radiotherapy), adjuvant drugs, and implementation of sub-type specific targeted therapies demonstrate no difference in local recurrence or survival for breast conserving therapy and mastectomy.

F Fitzal raised the necessity of surgery after neoadjuvant therapy (NACT) with surgical safety in new border i.e. confirming absence of microcalcifications [28] and attention being paid to residual scatter cells over original volume of the tumour. In patients N+ pre-NACT, SNB should be performed after the neoadjuvant treatment as in about 30% of cases the axilla becomes negative with treatment. This technique is feasible and accurate when surgical procedures are improved by the excision of two or more SLNs and by using two different detective techniques, like AUS, MRI, PET CT [29]. False negative rates can be decreased considering any size of metastasis (1, 1mi, is+) in the SLN after NAC sufficient to perform axillary lymph node dissection.

## Radiotherapy

Many physicians spoke about radiotherapy: T Whelan, T Bucholz, R Orecchia, J Jassem, and F Sedlmayer.

Since it was first introduced over 40 years ago, breast irradiation has evolved significantly especially owing to the advances in radiation, technology, surgery, and systemic therapy. High-risk patients (<50 yrs, G3, TNBC, close margins) should be approached in a standard way with WBI 50 Gy/25 fractions + boost irradiation of 10–16 Gy in 5–8 fractions of the tumour bed, whereas patients with other classes of risk could be treated with tailored therapy.

Based on the hypothesis that breast cancer cells are, compared to normal cells, equally sensitive to large doses per fraction in medium-risk patients, hypofractionation (a larger dose per fraction in a smaller number of fractions: 40–42.5 Gy in 15–16 fractions given over three weeks) appears to be the gold standard. In fact, if on one hand evidence shows similar results in local control and late toxicity, on the other hand it shows less acute side effects with improved quality of life. There are many new alternative approaches to partial breast irradiation limited to the surgical cavity plus a margin, as for example interstitial brachytherapy, Three dimensional conformal radiotherapy (3D-CRT), Balloon catheter brachytherapy, intraoperative radiation therapy (IORT), but it is still early for results.

In patients with low risk of RD, such as older women >65–70 yrs, ER+, low stage, and no lymph node involved, according to many pieces of data ( e.g. Tinterri, Fisher, Winzer, Blamey, Fyles; Kunkler, Hughes, Potter), the WBI should be considered as a treatment option and therefore WBI could probably be omitted.

The selection criteria for RT of regional lymph nodes are still being defined.

The role played by radiation in helping to achieve these favorable results could not be specifically assessed in this study. However, this trial led to a new standard of doing less axillary treatments for selected patients with lymph node positive breast cancer. A meta-analysis of MA.20 and EORTC2292-10925 suggested that the addition of RT to the level III axillary, supraclavicular, and upper internal mammary lymph nodes improved statistically-significant OS, DFS, and distant metastasis free survival. On the contrary, some studies suggested that patients with 1–3 positive lymph nodes treated with mastectomy and systemic therapy had no benefits from post-mastectomy RT that included regional lymph node RT. In addition, a US National Cancer Database study that evaluated regional lymph node RT in patients treated with breast conservation found no difference in OS after five years with the additional RT. Nowadays, the challenge is to apply identified biomarkers to the different BC subtype to predict the response to RT. For example, luminal A BC seems to have high radiosensitivity showing the lower rate of local regional recurrence (LRR) after RT. This is seen especially when combined with other features, such as older age, small tumour size, low or intermediate grade, and low Ki-67. Luminal B BC have intermediate radiosensitivity and intermediate LRR; HER2+ BC have low radiosensitivity and intermediate LRR; TNBC have very low radiosensitivity and high LRR. [30]

Mastectomy generally concerns patients with a more advanced stage of the disease and many of them will require post-mastectomy radiotherapy. Regarding this issue, J Jassem pointed out that breast reconstruction prior to RT, particularly using prosthetic material, is associated with increased risk of complications (capsular contraction, pain, and distortion) which may affect the final cosmetic effect. The optimal integration of breast reconstruction and RT should be based on the individual situation and on the informed patient preferences. Reconstruction post-mastectomy should not change RT dose, fractionation, and irradiate regions. It is true that reconstructed tissues or implants may compromise RT planning and impair treatment outcome, but this problem can be alleviated with the modern RT technique.

IORT seems to have an important role, and it is more established as a boost preceding WBI. At median follow-up periods of six years, significantly low local recurrence rates of less than 1% are observed. IORT seems to have immunological effects as well, including block of cell proliferation, induction of cytokines, and anti-angiogenic effects.

## Conclusions

This year the St Gallen Consensus Conference offered useful advice for the management of early breast cancer in women by integrating knowledge of earlier studies with data of recent studies thereby offering valuable insights on how best to proceed in scientific research.

A future in which immunotherapy and targeted therapy based on genetic signatures will be fundamental in clinical practice routine as the most effective drug in terms of precision medicine is becoming more and more real. This in association with surgical and radiation personalised treatments will make the fight against breast cancer ever more effective. This is with special attention to not only the quantity but also the quality of patients’ life.

Prof Umberto Veronesi once said: ‘Believing in science means believing in the future’, which was clearly the important message of this conference.
